# Experimental Investigation of the Blanked Surface of C5191 Phosphor Bronze Sheet over a Wide Range of Blanking Speeds

**DOI:** 10.3390/ma13153335

**Published:** 2020-07-27

**Authors:** Lei Wang, Daochun Hu, Minghe Chen, Hongjun Wang

**Affiliations:** 1 School of Mechanical Engineering, Nanjing Vocational University of Industry Technology, Nanjing 210023, China; 2018100944@niit.edu.cn; 2 Jiangsu Key Laboratory of Precision and Micro-Manufacturing Technology, Nanjing University of Aeronautics and Astronautics, Nanjing 210016, China; cmh@nuaa.edu.cn; 3 Engineering Technology Training Center, Nanjing Institute of Industry Technology, Nanjing 210023, China; whj@niit.edu.cn

**Keywords:** C5191 phosphor bronze, blanking process, blanked surface quality, adiabatic shear, dynamic recrystallization

## Abstract

The influence of blanking speed on the blanked surface quality of C5191 bronze phosphorus sheets, with a thickness of 0.12 mm, was systematically studied to demonstrate the mechanism under high speed blanking. The morphology and microstructure of the blanked edge were observed by using a variety of techniques, including optical microscopy (OM), scanning electron microscope (SEM), electron backscatter diffraction (EBSD), and transmission electron microscope (TEM). The results revealed that the local temperature and microhardness of the shear zone increased with the increase in blanking speed. Moreover, the quality of blanked edge significantly improved with the increase in blanking speed due to the combined influence of strain rate hardening and thermal softening. In addition, the blanked edge grains were elongated along the blanking direction and formed dislocation cells and sub-grains in some areas. The blanked edge is dominated by {000} <100> cubic texture at higher blanking speeds, and {112} <111> texture at lower blanking speeds. When punched at an ultra-high speed of 3000 strokes per minute (SPM 3000), the local area of the blanked edge exhibited distinct microstructural features, including low dislocation density, nanocrystals with high-angle grain boundaries, and significant differences in grain orientation. Additionally, the selected area electron diffraction (SAED) pattern exhibited a discontinuous ring-like structure, indicating the occurrence of adiabatic shearing with dynamic recrystallization.

## 1. Introduction

The blanking process is widely utilized in sheet metal forming in the automotive and electronics industries. However, the production process raises certain challenges due to the complexities of the blanking process. Moreover, the blanking speed should be increased to deal with the large-scale production and lower the labor cost. However, the higher blanking speed renders additional inertia, viscosity, and thermal effects, influencing the blanked edge profile and, in turn, the quality of the end product. The blanked edge profile exhibits distinct characteristics, including rollover zone, shear zone, fracture zone, and burr zone. In general, the proportion of shear zone is utilized as a quantitative indicator of the quality of the blanked edge.

Demmel et al. [1] studied the effect of blanking clearance and blanking speed on maximum blanking temperature by dynamically measuring the contact between punch and sheet. The results revealed that the blanking speed mainly influenced the increase in temperature, which should be considered in a process development and Finite Element Method (FEM) simulations to predict plastic fracture. It has been reported that higher blanking speeds are required to obtain deeper insights into the prediction of ductile fracture. Hu et al. [2] proposed a method to predict the temperature distribution during blanking by establishing a thermal-mechanical FEM model and characterized the consistency of the blanked edge at different blanking speeds. The results showed that high-speed blanking provides higher production efficiency without compromising the quality of the final product. Canales et al. [3] developed an FEM-based blanking process and studied the influence of punch speed on the quality of the blanked edge and characteristic blanking parameters, such as maximum blanking force, maximum displacement, and temperature variation.

Lubis et al. [4,5] demonstrated that a higher punching speed and smaller distance between blanking clearance increase the edge quality of the final product. Consequently, the burr height and rollover are reduced, whereas the shear zone ratio of the blanked edge is improved. Moreover, Wang et al. [6] and Fazily et al. [7] used optical microscopy (OM) and scanning electron microscopy (SEM) to analyze the characteristic size, fracture morphology, and strength of the shear edges, and investigated the surface microhardness of the shear zone (SZ) and shear-affected zone (SAZ) using Vickers hardness test.

Furthermore, several studies have focused on the macro-quality of the blanked edge and carried out numerical simulations to better understand the blanking process. However, the direct microstructural observation of the blanking process, carried out at different blanking speeds, has not been carried out due to technological constraints. 

Herein, the microstructural evolution of the blanked edge were studied through a series of blanking experiments, ranging from low blanking speed to high blanking speed. These results provide novel insights into the formation mechanism of the blanked edge and facilitate the development of high-speed blanking processes for industrial applications.

## 2. Experimental Procedures

C5191-H bronze phosphorus (Kunshan Sanbao Cooper Co., Ltd., Kunshan, China), with a nominal composition (wt. %) of Zn (5.5–7.0), P (0.11–0.13), Fe (≤0.02), Pb (≤0.05), Zn (≤0.20), and otherwise Cu, was used as a blanking specimen. The thickness and width of the C5191-H bronze phosphorus specimen were 0.12 mm and 24 mm, respectively.

**Figure 1:**
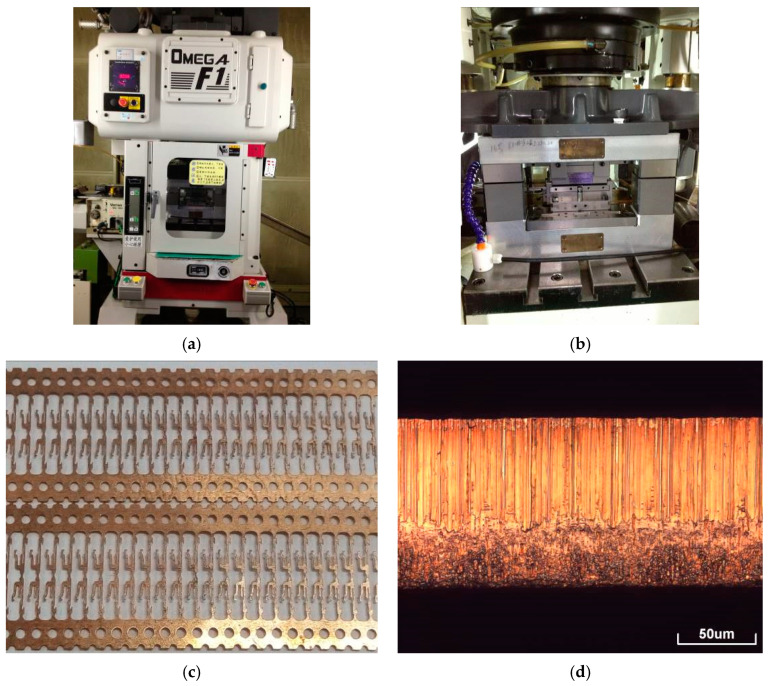
Ultra-high-speed punching press and ultra-high-speed precise progressive stamping die. (**a**) DOBBY-OMEGA F1 ultra-high-speed punching press; (**b**) Ultra-high-speed precise progressive blanking die; (**c**) Thin sheet strips after ultra-high-speed blanking; (**d**) Morphology of thin sheet blanked edge after ultra-high-speed blanking.

The thin sheets high-speed blanking experiment was carried out at Kunshan Jiahua Electronics Co., Ltd., China. The ultra-high-speed precise progressive stamping die, developed through the National Torch Plan Project, has a maximum punching speed of up to 3000 strokes per minute (SPM 3000). The ultra-high-speed punching press, die, and strips are shown in Figure 1. The blanking speeds of SPM 300, SPM 600, SPM 800, SPM 1200, SPM 1500, and SPM 3000 correspond to 58 mm/s, 175 mm/s, 350 mm/s, 465 mm/s, 700 mm/s, 875 mm/s, and 1750 mm/s, respectively. Moreover, at each blanking speed, the strips (1000 mm) were sectioned after continuously blanking for 1 h for subsequent characterization.

A high-magnification optical microscope (OM, Olympus GX71, Olympus Corporation, Shinjuku, Tokyo, Japan) and microhardness tester (Shimadzu HMV-20, Shimadzu Corporation, Nakagyo-ku, Kyoto, Japan) were used to obtain the profile image of the blanked edge and microhardness of different zones. The microstructure was examined by using a scanning electron microscope (SEM, JSM-6360 LV/JEOL, Japan Electronics Co., Ltd, Toyoshima, Tokyo, Japan), equipped with electron back-scattered diffraction (EBSD) mode and a transmission electron microscope (TEM, JEM-2100F/JEOL, Japan Electronics Co., Ltd, Toyoshima, Tokyo, Japan). Prior to microstructural observations, the samples were ground and polished, and TEM samples were prepared by ion thinning. 

## 3. Results and Discussion

### 3.1. Influence of Blanking Speed on Blanked Edge Quality

The height of the rollover zone is not in an order of magnitude compared with that of shear or fracture zones, which can be ignored. Moreover, the borders between the shear and fracture zones are irregular contour lines and cannot be considered straight. Hence, the statistical method, which considers the average value of the shear zone height at different positions, cannot accurately and truly reflect the quality of the blanked edge. Therefore, the proportion of shear zone as a characteristic parameter of the blanked edge quality to reduce the measurement error was considered. The specific characterization process can be given as follows.

**Figure 2:**
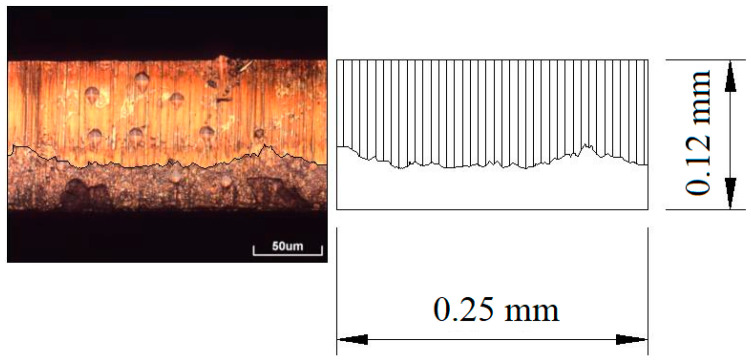
Schematic illustration of the blanked edge quality evaluation.

First, high-magnification OM and SEM were used to collect the profile image of the blanked edge. Second, the image was imported into AUTOCAD at a ratio of 1:1. Then, the shear zone area was computed by using the “Region Area” command in the AUTOCAD software. Finally, the ratio between the shear zone area (A_i_) and the blanked edge area (A_0_) was calculated, as shown in Figure 2.

**Figure 3:**
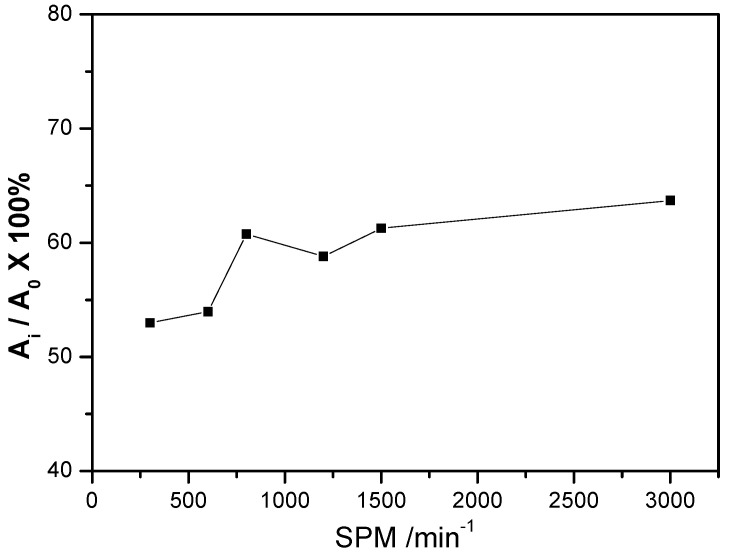
Influence of the blanking speed on blanked edge quality of phosphor bronze sheet.

The influence of the blanking speed on blanking quality was assessed by using the blanked edge quality characterization method, as shown in Figure 3.

Hence, the quality of the blanked edge is significantly improved with the increase in blanking speed. Addition, the proportion of shear zone increased from 52.98% to 63.69% when the blanking speed was increased from SPM 300 to SPM 3000. Owing to the high blanking speed, the strain rate hardening led to the brittle fracture of the thin sheets. Moreover, the proportion of shear zone decreased with increasing proportion of the fracture zone, compromising the quality of the blanked edge. At the same time, the higher blanking speed increased the localized temperature, which improved the sheet toughness and delayed brittle fracture. The proportion of the fracture zone decreased with the increasing proportion of the shear zone, which improved the quality of the blanked edge. It can be concluded that the influence of higher blanking speed on localized temperature exceeded the strain rate hardening effect, which resulted in improved quality of the blanked edge.

### 3.2. Influence of Blanking Speed on Temperature Distribution

#### 3.2.1. Microhardness distribution

**Figure 4:**
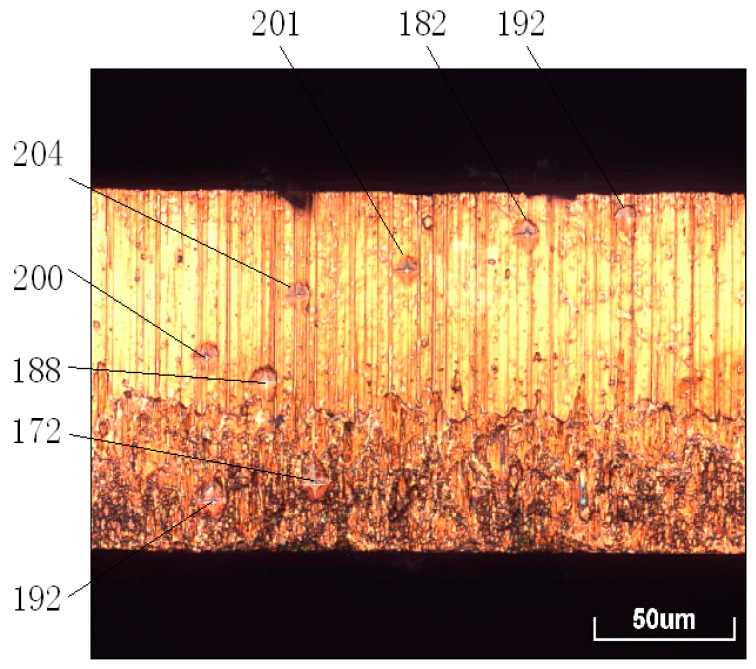
Microhardness of the blanked edge of phosphor bronze sheet (Units: HV).

A Shimadzu HMZ-G20 microhardness tester (Figure 4) was employed to investigate the microhardness of the blanked edge at different blanking speeds. The results are shown in Table 1.

Figure 4 shows that the microhardness started to increase from the border of the shear zone and attained a maximum value in the middle. Then, the microhardness gradually decreased and attained a minimum value at the boundary between the shear and fracture zones. A slight increase in microhardness is also observed in the fracture zone, which can be ascribed to the thermal effect in the blanking process.

Table 1 shows that the microhardness of the boundary between the shear and fracture zones gradually decreased with increasing blanking speed. As mentioned earlier, the thermal softening effect surpassed the strain hardening effect with increasing blanking speed. Hence, the center of the shear zone rendered a lower microhardness than the edges. One should note that the microhardness distribution in the blanked edge is consistent with the temperature distribution, as described by Hu et al. [2], rendering a “spindle shape” pattern and indicating the similarity of the influence trend between microhardness and temperature.

#### 3.2.2. Temperature Distribution

The deformation of materials under high strain rates can be regarded as an adiabatic process. According to Kapoor et al. [8], 10% of the plastic deformation work is stored inside the system, while 90% of the plastic deformation energy is transformed into thermal energy, which raises the temperature of the system (Δ*T*) as given below:

(1) $$\Delta T=(0.9\rho_{0}{}^{-1}C_{v}{}^{-1})\int_{0}^{\varepsilon_{T}}\sigma_{s}d_{\varepsilon_{s}}$$

where $\rho_{0}$ and $C_{v}$ represent the density and specific heat capacity of the material, respectively.

**Figure 5:**
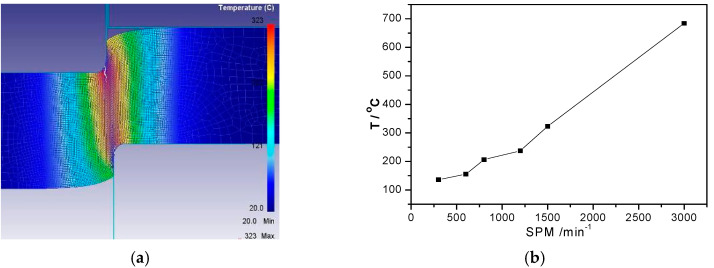
FEM-simulated temperature distribution of the blanked edge at different blanking speeds. (**a**) Temperature distribution of blanked surface 1500 strokes per minute (SPM 1500); (**b**) Relation curve between temperature distribution of blanked surface and blanking speed.

Equation (1) indicates that the temperature of the blanked edge linearly increases with increasing strain rate. It has been reported that the FEM simulations can predict the temperature distribution of the blanked edge at different blanking speeds [2], as shown in Figure 5.

Overall, the temperature of the blanked edge increased significantly with increasing blanking speed. For instance, when the blanking speed was increased from SPM 1500 to SPM 3000, the local temperature increased from 323 °C to 684 °C, respectively, exceeding the dynamic recrystallization temperature of C5191 phosphorous bronze (188.3–254.2 °C) and, consequently, resulting in thermal softening and dynamic crystallization. However, as the blanking deformation is completed in a certain time, the heat generated by plastic deformation cannot be completely diffused from the deformation zone to the surrounding substratum, increasing the local temperature and resulting in the thermal softening effect; hence, thermo-plastically instability occurred, in agreement with the results of Rafsanjani et al. [9].

### 3.3. Microstructural Analysis of Blanked Edge

#### 3.3.1. EBSD Analysis

**Figure 6:**
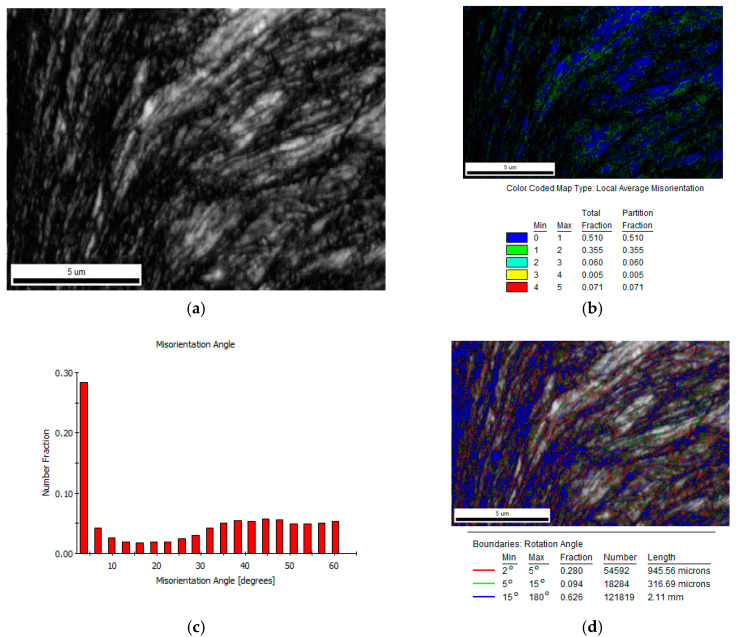
Orientation diagram of deformed zone of blanked edge (SPM 600). (**a**) Image quality (IQ) map; (**b**) Local average misorientation (LAM); (**c**) Misorientation angle distributions; (**d**) Grain boundaries map.

The ESBD can provide texture characteristics of the blanked edge and adjacent area and demonstrate the microstructural evolution during the blanking process. Figure 6a presents the EBSD image quality (IQ) of the blanked edge and different gray levels in which images reflect the grayscale quality of the Kikuchi line. One should note that the residual strain distorts the crystals and lowers the quality of the diffraction pattern. According to Huang et al. [10], the IQ can qualitatively represent the strain distribution within the microstructure. The plastic deformation causes a large number of dislocations in the metal grains, and lattice distortions accumulate around the dislocations.

The highly deformed material locally alters the orientation of small angles and forms different substructures, such as dislocation walls, microstrips, and sub-grains. Cao et al. [11] and Humphreys et al. [12] stated that the orientation changes represent the degree of dislocation accumulation under severe deformation. According to Meng et al. [13], it is worth emphasizing that the local average orientation difference (Figure 6b), which can be obtained from the EBSD analysis, is highly sensitive to small orientation changes and can be used to estimate dislocation density. Figure 6c,d show the distribution of grain orientations and grain boundaries in the deformed area of the blanked edge. During the blanking process, the deformed area of the blanked edge produces a large number of geometrically necessary boundaries (GNBs) and incidental dislocation boundaries (IDBs), rendering severe plastic deformation to the grains in the given area. The grains are elongated along the blanking direction, resulting in a large difference in grains’ orientation and forming a layer of grain fragmentation. Moreover, a high proportion of high-angle GNBs, coordinating different strain regions, evolved into grain boundaries. In general, the blanking process rotates the grains to obtain uniform and preferred grain orientations.

**Figure 7:**
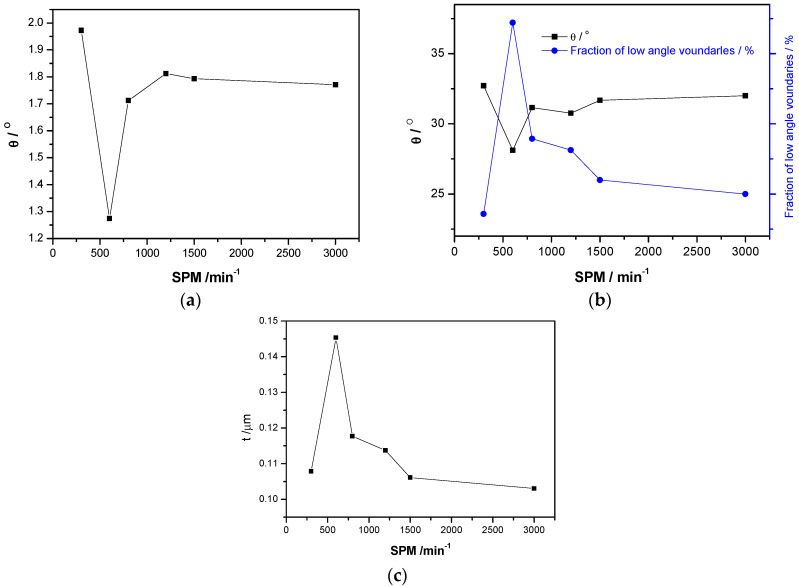
Electron back-scattered diffraction (EBSD) maps of the blanked edge prepared under different blanking speeds. (**a**) Local average orientation difference; (**b**) Grains misorientation; (**c**) Grain size.

Furthermore, the EBSD analysis of the blanked edge, prepared under different blanking speeds, was also carried out, and results are shown in Figure 7. At lower blanking speeds, the local average orientation difference and grain orientation were found to be more significant (SPM 300 and one more speed), whereas the grain size decreased and the difference in local orientation and grain orientation became less significant with increasing blanking speeds, such as SPM 1200, SPM 1500, and SPM 3000.

**Figure 8:**
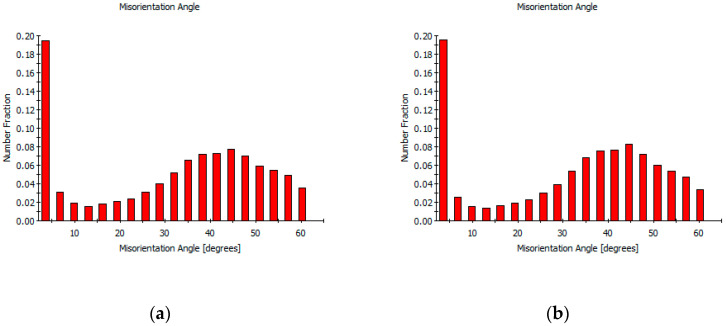

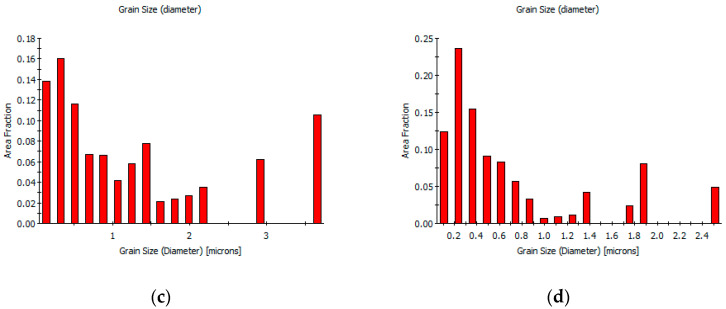
Differences in grain orientation and grain size of blanked edge under high speed blanking. (**a**) Misorientation angle of blanked surface at SPM 1500; (**b**) Misorientation angle of blanked surface at SPM 3000; (**c**) Grain size of blanked surface at SPM 1500; (**d**) Grain size of blanked surface at SPM 3000.

The differences in grain orientation and grain size under high-speed blanking are shown in Figure 8. The proportion of the high-angle and small-sized grains significantly increased with increasing blanking speed. Hence, the dislocation density decreased in the local region of the blanked edge under ultra-high blanking speed. Moreover, the local average orientation difference became smaller and micro-crystals of sub-micron grain size were formed with increasing blanking speed. In addition, a high number of high-angle interfaces, with a certain degree of preferred grain orientations, i.e., distinctive texture, were observed after the high-speed blanking.

**Figure 9:**
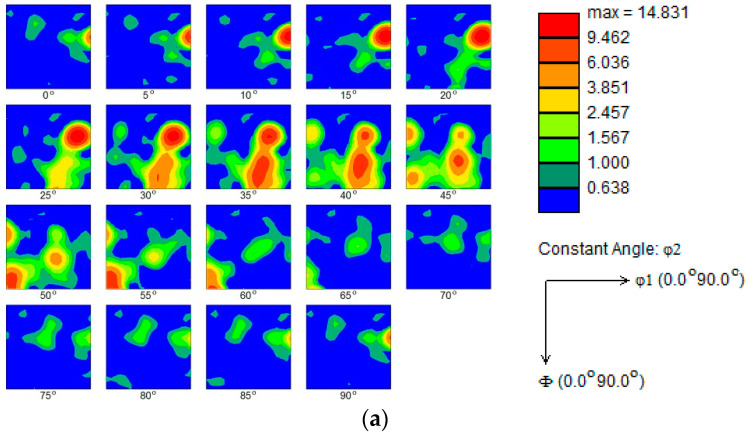

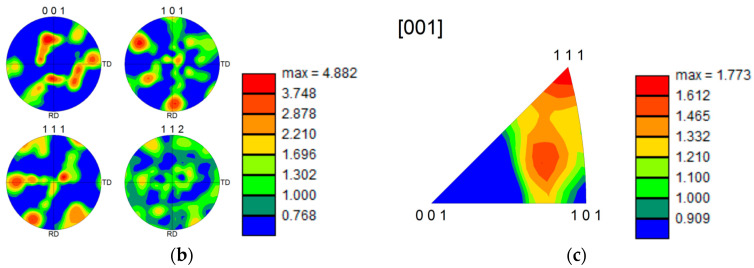
Texture pattern of the deformed zone in the blanked edge (SPM 3000). (**a**) Orientation distribution function (ODF) map; (**b**) Pole Figure map; (**c**) Inverse Pole Figure map.

Bunge et al. [14] defined the Euler angle, which describes that any crystal orientation can be achieved by rotating from the initial orientation in the order of φ1, Φ, and φ2. By defining φ1, Φ, φ2 = 0 to 90°, an orientation distribution function (ODF) can be constructed by selecting Euler orientation feature maps of φ2 with an interval of 5°. The ODF represents the texture information of the diffractive microregion, which can be combined with the EBSD-derived pole figures and reverse pole figures to obtain the texture characteristics of the blanked edge, as shown in Figure 9.

INCA Crystal software was utilized to analyze the texture of the blanked edge. At low blanking speeds, i.e., SPM 300 and SPM 600, a relatively stronger {112} <111> Cu texture, with corresponding pole density of 13 and 10, and a weaker {221} <1-46> Cu texture, with corresponding pole density of 9 and 5, are formed in the blanked edge. On the other hand, at relatively higher blanking speeds, i.e., SPM 1500 and SPM 3000, a relatively stronger {001} <100> cubic texture, with corresponding pole density of 9 and 12, and a weaker {011} <0-11> Cu texture, with corresponding pole density of 4 and 7, are formed in the blanked edge. It has been reported that the cubic oriented grains have more advantages in nucleation quantity and grain size than other orientaions. Chen et al. [15] and Xu et al. [16] concluded that the cubic textures are formed during the recrystallization annealing, which leading to nucleation and high-angle grain boundary migration. Therefore, the {001} <100> cubic texture is preferentially formed in the blanked edge under high blanking speeds, whereas the {112} <111> rolling texture is dominant under low blanking speeds.

#### 3.3.2. TEM characterization

**Figure 10:**
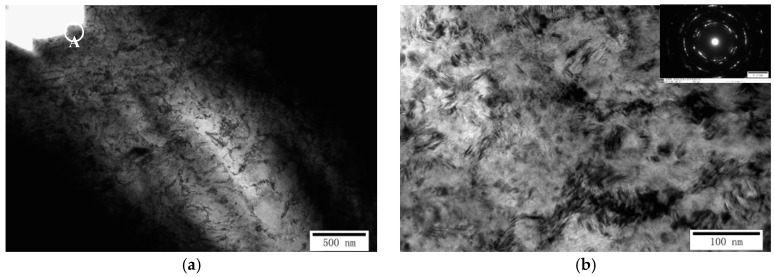
Transmission electron microscope (TEM) image and selected area electron diffraction (SAED) pattern of the blanked edge and adjacent deformation zone (SPM 1200). (**a**) TEM image of the vertical blanked edge (I); (**b**) TEM image and SAED pattern of the selected area.

As mentioned earlier, the vertical blanked edge (I) specimen was prepared for TEM characterization to observe the microstructural evolution of the blanked edge and surrounding deformation area, as shown in Figure 10. It can be seen that the blanked edge and adjacent deformation zone render different microstructural characteristics. In the zone adjacent to the blanked edge (Area-A), grains are severely elongated along the blanking direction, and large-deformation grains are generated due to the combined effect of pressure and shear stress. When grains elongate and form elongated thick-walled dislocation cells, the dislocations are further multiplied and entangled to form dislocation walls. Hence, the dislocation walls collapse into multiple smaller sub-grains with small orientation differences under the influence of the thermal softening effect. Under blanking deformation, smaller equiaxed regions are formed by the rotation of sub-grain boundaries, and heterogeneous dislocations, present on the sub-grain boundaries, disappear with the progression of the deformation process, as described by Alawadhi et al. [17]. Hence, several ultra-fine grains in Area-A with the grain size of 50–100 nm were observed. Moreover, the selected area electron diffraction (SAED) exhibited a clear ring-like pattern, which is consistent with the EBSD results. Hence, based on the obvious grain boundaries and low dislocation density, it can be concluded that some ultra-fine grains contain typical dynamically recrystallized grains, which was in agreement with the results of Tomczyl et al. [18].

**Figure 11:**
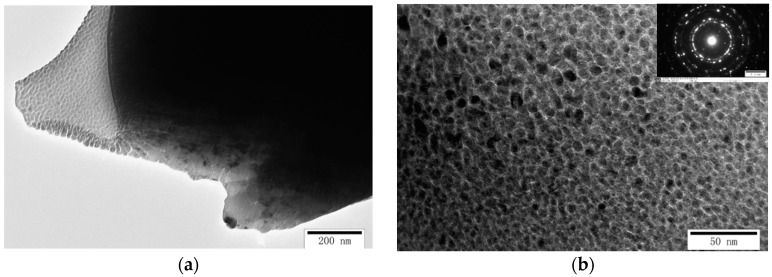
Microstructure of the Area-A of the blanked edge prepared under ultra-high blanking speed (SPM 3000). (**a**) TEM morphology; (**b**) Nano-grains.

Based on the rotational dynamical recrystallization (RDR) theory, proposed by Nesterenko et al. [19], the microstructure of the ultra-high-speed blanked edge is influenced by several factors, such as immense strain, high plastic strain rate, and temperature. Hence, dislocation cells and dislocation entanglements continuously absorb dislocations and transform into sub-grains/grain structures, creating undesirable sub-grains boundaries. The sub-grain boundaries are formed due to the short-range movement of high-angle grain boundaries, and sub-grains are elongated, broken, and rotated due to ultra-high strain, resulting in the formation of nano-grains (Figure 11b).

Moreover, the diffraction pattern of the recrystallization area shows complete and smooth diffraction rings, exhibiting a large orientation difference between grains, which are no longer sub-grains and exhibit high-angle grain boundaries. According to Li et al. [20], in accordance with the thermal softening effect, severe localized plastic deformation occurred, which may produce adiabatic shear and introduce dynamic recrystallization characteristics.

**Figure 12:**
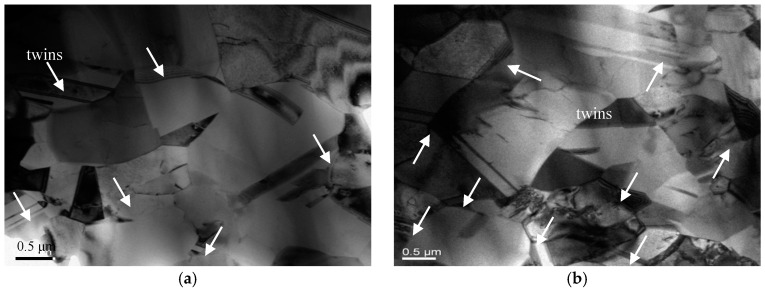
TEM images of the shear zone at the blanking speed of (**a**) SPM 300 and (**b**) SPM 3000.

A cross-sectional TEM specimen was prepared to further explore the microstructural evolution of the blanked edge near the shear zone, and results are shown in Figure 12. One should note that the grain size of the shear zone in sample II is significantly larger than the grain size of the shear zone in sample I, which is perpendicular to the blanked edge near the shear zone, indicating the elongation of grains along the blanking direction. Moreover, unique deformation twins are formed in the shear zone of the blanked edge, which can be ascribed to the difficulty of slipping. As C5191 phosphor bronze belongs to the face-centered cubic system, it possesses several slip systems and exhibits slipping deformation. However, the dislocation density increases significantly during the high-speed blanking process because the blanked edge is momentarily subjected to massive shear stress, and dislocations entangle with each other to form dislocation walls, hindering the initiation of the slip system. The presence of twins compensates for the lack of slipping deformation and satisfies the demand for severe deformation as demonstrated in Zhou et al. [21]. In Figure 12, the twin interface is not a straight line and exhibits a certain degree of curvature, indicating extreme lattice distortions around the twin boundaries and occurrence of polygonization inside and around the twins, in line with the results of Yang et al. [22] and Ryabov et al. [23]. With increasing blanking speed, more dislocation movements are generated inside the grains. However, the grain boundaries block these movements; consequently, a large number of dislocations accumulate near the grain boundaries and form the sub-grain structures. When the blanking speed is further increased, these newly formed sub-grains form high-angle grain boundaries (Figure 12b).

During the blanking process, dislocations gradually tangle together to form dislocation cells. As the blanking deformation continues, the number of dislocation cells increases and the size of dislocation cells decreases. When the dislocation density in the cell walls further increases, dislocations continuously move towards the grain boundaries. Finally, the dislocation cells collapse due to the constant gathering of the dislocation entanglement in the cell walls, resulting in the formation of a small-angle structural interface. Within the grains, a large number of unstable sub-micron sub-grains appear, and small-angle textures rotate under large deformation blanking. Thereby, twins and sub-grains are broken, squeezed, and twisted with each other. A large number of grain defects are entangled and form high distortion energy. Under the influence of thermal softening, sub-grains gradually coarsen and form large-angle grain boundaries. These grains continue to grow as nuclei during recrystallization, and the final grains exhibit a significantly larger size than the grains in the sample’s area I.

## 4. Conclusions

(1) The blanked edge quality of C5191 phosphor bronze sheet is significantly improved with increasing blanking speed. For instance, the proportion of shear zone in the blanked edge increased from 52.98% to 63.69% with an increase in blanking speed from SPM 300 to SPM 3000, respectively.
(2) Moreover, deformation twins were observed in the shear zone of the blanked edge, which rotated and became coarser under the influence of blanking extrusion and thermal softening. Hence, new sub-grains are formed with clear high-angle grain boundaries. Furthermore, during ultra-high-speed blanking, partial adiabatic shear occurred in the blanked edge due to high-speed deformation and changes in adiabatic temperature, which was accompanied by dynamic recrystallization and improved the quality of blanked parts.
(3) Due to its unique dynamic deformation mechanism, C5191 phosphor bronze can significantly improve the quality of blanked surface and meet the needs of low-cost mass production.

## Figures and Tables

**Table 1:** The microhardness (HV) of the blanked edge at different blanking speeds.

Blanking Speed	Shear and Fracture Zones Boundary	Shear Zone
(Matrix microstructure)	210	210
SPM 300	194	216
SPM 600	196	214
SPM 800	192	199
SPM 1200	188	203
SPM 1500	171	218
SPM 3000	168	239
